# Preventive effects of a novel herbal mixture on atopic dermatitis-like skin lesions in BALB/C mice

**DOI:** 10.1186/s12906-018-2426-z

**Published:** 2019-01-18

**Authors:** Abraham Fikru Mechesso, Seung-Jin Lee, Na-Hye Park, Jin-Yoon Kim, Zi-Eum Im, Joo-Won Suh, Seung-Chun Park

**Affiliations:** 1 Laboratory of Veterinary Pharmacokinetics and Pharmacodynamics (LVPP), College of Veterinary Medicine, Kyungpook National University, 41566, 80 Daehakro, Bukgu, Daegu, Republic of Korea; 2 Forest Resources Development Institute of Gyeongsangbuk-do, Andong, Gyeongsangbuk-do 36605 Republic of Korea; 3 Center for Nutraceutical and Pharmaceutical Materials, Division of Bioscience and Bioinformatics, Science campus, Myongji University, 449-728 Yongin, Gyeonggi Republic of Korea

**Keywords:** Atopic dermatitis, Inflammation, Mice, Skin

## Abstract

**Background:**

A combination of parts of *Cornus officinalis, Rosa multiflora, Lespedeza bicolor*, *Platycladus orientalis*, and *Castanea crenata* is commonly used for alleviating inflammatory skin disorders. Therefore, this study was carried out to evaluate the in vitro and in vivo preventive effects of a novel herbal formula made from the five plants (C2RLP) against atopic dermatitis in BALB/C mice.

**Methods:**

Mice were allocated into five groups (*n* = 8) including, control (Normal, petrolatum, and betamethasone treated) and treatment groups (treated with 2.5 and 5% C2RLP ointment). Atopic lesion was induced by applying 1-Chloro-2, 4-dinitrobenzene to the dorsal thoracic area of mice. Macroscopical and histological evaluations were performed to determine the effects of treatment on the progress of the skin lesions. The effects of treatment on the production and release of interleukins, interferon -ϒ, nitrite, prostaglandin E2, thymus and activation-receptor chemokine, and β-hexosaminidase were evaluated and comparisons were made between groups. In addition, the chemical compounds present in C2RLP were identified by Liquid Chromatography-Mass Spectrometry.

**Results:**

Topical application of C2RLP reduced the dermatitis score and suppressed histopathological changes in mice. Treatment significantly reduced (*P* < 0.05) plasma IL-4 level, the production of nitrite, prostaglandin E2, and thymus and activation-receptor chemokine production. The lipopolysaccharide-induced iNOS-mRNA expression in RAW 264.7 cells was also suppressed by high concentrations of C2RLP. In addition, C2RLP showed an inhibitory effect against DPPH free radical (IC_50_ = 147.5 μg/ml) and β-hexosaminidase release (IC_50_ = 179.5 μg/ml). Liquid Chromatography-Mass Spectrometry analysis revealed the presence of various compounds, including loganin, ellagic acid, and kaempferol 3-glucoside.

**Conclusion:**

Down-regulation of T- helper 2 cellular responses and suppression of inflammatory mediators contributed to the protective effects of C2RLP from atopic dermatitis in BALB/C mice.

## Background

Atopic dermatitis (AD) is an allergic skin disease characterized by complex symptoms such as drying and thickening of the skin, and scratch marks that are frequently associated with immunoglobulin-E (IgE) hyper-responsiveness to environmental allergens. The wrist, neck, face, and the crooks of the elbows and knees are among the most frequent locations of the lesions [1]. AD is mostly affecting children with onset before the age of five years [2]. Environmental (house dust mites and air pollution) and genetic factors considered the causes of AD. In addition, genetic predisposition accompanied by assorted peculiar immune symptom accounts for more than 50% of reported cases [3].

Activation of T-helper 2 (Th2) and mast cells are mentioned in the development of AD [4]. It is associated with an increase in serum concentration of Th2 cytokines, including interleukin (IL) -4, IL-5, IL-10, and IL-13. In addition, expression of interferon- γ (IFN-γ) is also reported in cases of AD [5, 6]. Basal keratinocytes produce thymus and activation-regulated chemokine (TARC) recruits Th2-lymphocytes and further aggravate dermatitis [7]. Authors also suggested that the release of β-hexosaminidase from degranulated mast cells, high levels of serum immunoglobulin (Ig)E, and the expression of proinflammatory mediators such as prostaglandin E2 (PGE2) and nitrite (NO) are important determinants in the propensity of mice to AD [8, 9].

In spite of the profound side effects caused by topical steroids and oral anti-histamines, these drugs are commonly used to treat AD [10]. Hence, efforts have been directed towards identifying safer and effective compounds of plant origin which can modulate the pathological mechanism(s) of AD such as anti-histamine effects, inhibition of Th_2_ responses and IgE production [11]. More than 20% of the population in Korea rely on traditional medicine as the primary health care [12]. However, only a few studies have been conducted on the efficacy and safety of medicinal plants. Therefore, a screening test was conducted using the DPPH antioxidant, β-hexoseaminidase, and NO assay on 286 plants from Gyeongbuk Forest Resource Development Institute, Republic of Korea. These assays were selected as a screening method, taking into account the multifactorial nature of AD. Accordingly, *Cornus officinalis* (Family: Cornaceae), *Castanea crenata* (Family: Fagaceae), *Rosa multiflora* (Family: Cornaceae)*, Lespedeza bicolor* (Family: Legumes), and *Platycladus orientalis* (Family: Cupressaceae) were selected for further studies. Previous studies have shown that these plants produced various degrees of biological activities that are associated with AD [13–17]. In addition, various parts of these plants combined in different proportions to produce ointments for inflammatory skin disorders, including AD [18–23]. However, all of the studies are conducted on the activities of a single plant against inflammation or free radical activity. To the best of our knowledge, there is no scientific report available on the efficacy and safety of the most commonly used plant combinations.

Based on the results of the screening assay, a 4:1:1:1:1 ratio of *Cornus officinalis* (fruit): *Rosa multiflora* (stem)*, Lespedeza bicolor* (aerial part), *Platycladus orientalis* (leaves), *Castanea crenata* (leaves) respectively, were selected for further studies. Additional investigation using the NO and β-hexosaminidase assay have demonstrated that C2RLP produced better activity than each plant extract (data not shown). Finally, a topical ointment was formulated (C2RLP) taking into account the main complaints of the disease such as pruritus, dryness, and psoriasis on the skin [24]. Therefore, the study was aimed to evaluate the in vivo protective effects of topical application of herbal formulation, C2RLP, against 1-Chloro-2, 4-dinitrobenzene (DNCB) induced AD-like lesion in BALB/C mice. In addition, the effects of C2RLP on free radical scavenging activity and cellular mediators were evaluated using various in vitro methods.

## Methods

### Plant extraction and preparation of ointments

*Rosa multiflora* (stem)*, Lespedeza bicolor* (aerial part), *Platycladus orientalis* (leaves), *Castanea crenata* (leaves) and *Cornus officinalis* (fruit) were purchased from the Gyeongbuk Forest Resource Development Institute, the Republic of Korea. The identity of the plants was confirmed by a taxonomist (Dr. Zi-Eum Im) and voucher specimens were deposited (LVPPM 2001–2005) in our laboratory. The dried and crushed parts of each plant were boiled in 30% ethanol (100 g/Liter). The extracts were filtered with Whatman filter paper Number 1 (GE Healthcare, UK Limited, UK), evaporated to dryness, and freeze-dried. A 5% (*w*/w) and 10% (w/w) ointments of C2RLP were made using petrolatum (Sigma-Aldrich) as a vehicle. For in vitro experiments on various cell lines, the extract was dissolved in the respective media used to grow the cells and then filtered using a 0.22-μm syringe driven filter (Merck Millipore Ltd., Carrigtwohil, Ireland).

### Experimental animals and materials

The availability of genetically manipulated strains, ease of manipulation, and low cost of mice as compared with other species of animals makes mouse models preferable to study AD. Therefore, specific-pathogen-free male BALB/C mice of 5 weeks old (male with an average weight of 18.5 g) were purchased from Orient Co. Seoul, South Korea (Charles River Technology) and acclimatized for 10 days. Mice were maintained in the animal room with 20–25 °C temperature, 55 ± 10% relative humidity, and 12 h light/dark cycle. A standard pellet diet and filtered tap water were given ad libitum. The total sample size (*n* = 40) was calculated using the G*power program based on α error probability of 0.05 and power (1- β error probability) of 0.80. The experiment was approved by the Institutional animal care and use committee of Kyungpook National University, Republic of Korea (KNU 2016–120). All experimental procedures were conducted according to the international guidelines for the care and use of laboratory animals [25].

### In vitro experiments

#### Cell culture

Murine macrophage RAW 264.7 cells, Rat Basophilic Leukemia cells (RBL-2H3), and human keratinocyte HaCa-T cells were obtained from the Korean Cell Line Bank, Seoul, and the Republic of Korea. RPMI medium (Roswell Park Memorial Institute) was used to maintain RAW 264.7 and RBL-2H3. The human keratinocyte HaCa-T cells were maintained in Minimum Essential Medium (MEM). Penicillin (100 U/ml), streptomycin (100 μg/ml), and fetal bovine serum (10% FBS) were added and incubated at 37 °C in 5% CO_2_ incubator. The RPMI, MEM, penicillin, streptomycin, and FBS used to supplement the medium were purchased from Sigma.

#### Measurement of NO and PGE2 production

RAW 264.7 cells (2 × 10^5^/ml) were cultured on 24-well plate and allowed to adhere to 80% confluence. Cells were treated with lipopolysaccharide (LPS, 0.5 μg/ml) for 30 min and incubated with C2RLP (10–300 μg/ml) for 18 h. NO production in the supernatant was determined using a spectrophotometer at 540 nm (VERSA max, Molecular Devices, Sunnyvale, CA, USA) and quantified from a standard curve generated using sodium nitrite (Griess Reagent System, Promega Co., Madison, WI, USA). Whereas, the level of PGE2 in the supernatant was measured using an Enzyme-linked immunosorbent assay (ELISA) kit (PGE2 ELISA kit, Cayman Chemical Co., Ann Arbor, MI, USA). In addition, the 3-(4,5-dimethyl-2-thiazolyl) -2,5-diphenyl-2H-tetrazolium bromide (MTT) assay was conducted to evaluate the effects of C2RLP on cellular viability following incubation of cells (2 × 10^5^/ml) with various concentrations of C2RLP for 24 h.

#### Evaluation of the effect of C2RLP on the expression of iNOS-mRNA

Total RNA was extracted from RAW cells using Trizol reagent (Invitrogen, Carlsbad, CA, USA). For cDNA synthesis, 1 μg of the RNA was subjected to RT reaction and amplified in triplicate using an Accu Power ® RTPreMix and ®PCR PreMix, respectively (Bioneer, Daejeon, Korea). The primer sequences used for amplification are summarized as follows: 5’-GTGGGCCGCCCTAGGCACCAG-3′ (F) and 5’-GGAGGAAGAGGATGCGGCAGT-3′(R) for β-actin; and 5’-CCCTTCCGAAGTTCTGGCAGCAGC-3′ (F) and 5’-GGGTGTCAGAGCCTCGTGGCTTTGG-3′ (R) for iNOS. A thermal cycler system (MyCycler, Bio-Rad Laboratory, and USA) was adjusted to the following reaction conditions. Initial denaturation and enzyme activation at 95 °C for 5 min, 35 cycle amplification at 95 °C for 45 s (denaturation), 60 °C for 45 s (annealing), and 72 °C for 45 s (extension).

#### Measurement of TARC production

The inhibitory effect of C2RLP on tumor necrosis factor-α and IFN-γ (TI) (Sigma-Aldrich) induced TARC production in HaCa-T cells were evaluated in accordance with the method of Lim et al. [26]. HaCa-T cells (1 × 10^6^/ml) were cultured on 24-well plates and stimulated with TI. The amount of TARC produced after 24 h following treatment with C2RLP was measured using an ELISA kit (R&D Systems Inc., Minneapolis, MN, USA). In addition, the cytotoxicity of C2RLP on HaCa-T cells was assessed using the MTT cell proliferation assay in the presence and absence of TI (10 ng/ml, each).

#### β-Hexosaminidase release assay

The effects of C2RLP on β-hexosaminidase release in RBL-2H3 cells was evaluated with some modifications of the method used by Kuehn et al. [27]. Briefly, RBL-2H3 cells (4 × 10^5^ cells/ml) were cultured on 24-well plate and incubated at 37 °C in 5% CO_2_ for 24 h. Following sensitization with anti-dinitrophenyl-immunoglobulin E (anti-DNP IgE) (Sigma-Aldrich) (100 ng/ml), the cells were washed (3x) and re-suspended in Siraganian buffer. An aliquot of 100 μL cells with C2RLP (10–300 μg/ml) was made into 96 well plates. Quercetin (Sigma-Aldrich) was used as a positive control. Following 30 min of incubation at 37 °C, cells were stimulated with 10 μL of 100 ng/ml of DNP-HAS (Sigma-Aldrich). The reaction was terminated by spinning the plate at 450 xg, at 4 °C for 5 min. An aliquot of 100 μL 1 μg/ml of N-acetyl-β-D-glucosamide (PNAG) (Sigma-Aldrich)) solution in citrate buffer (pH 4.5) was made into two new 96 well plates to measure the level of secreted and total β-hexosaminidase. Accordingly, 50 μl supernatant and 50 μl cell lysates were transferred to the plates containing PNAG solution and incubated for 90 min at 37 °C. The appearance of yellow color following the addition of 50 μL of 0.4 M Glycine buffer indicated the degree of β-hexosaminidase activity. Finally, optical density (OD) was measured at 405 nm and percentage β-hexosaminidase release was determined as follows.

% release = 100X [2(*A*-*B*))/ (1/2(*C*-*B*) + (4X (*D*- *B*)].

Where A is the OD value of the supernatant, B is the OD value of the plate blank, C is the OD value of the total supernatant, and D is the OD value of the lysates.

#### Antioxidant activity of C2RLP

The antioxidant effect of C2RLP against DPPH free radical was conducted following a previously described method [28]. The optical density was determined at 517 nm using a multichannel spectrophotometer (VERSA max, Molecular Devices, Sunnyvale, CA, USA) and the percent inhibitory effects of the test extracts on DPPH free radicals was calculated as follows:

$$ \mathrm{Inhibition}\left(\%\right)=100-\left[\frac{{\left(X\hbox{--} Y\right)}^{\ast }}{Z}100\right] $$

Where, X is the OD value of C2RLP with DPPH, Y is the OD value of C2RLP in ethanol, and Z is the OD value of Ethanol with DPPH.

#### Compound analysis of C2RLP using liquid chromatography-mass spectrometry (LC-MS)

LC-MS analysis of C2RLP was conducted by using an Accela UHPLC system (Thermo Fisher Scientific, CA, and USA) coupled with an LTQ-Orbitrap XL hybrid mass spectrometer (Thermo Electron, Bremen, Germany) via an ESI interface. Sample separation was carried out at room temperature using Waters BEH C18 column (2.1 × 150 mm, 1.7 μm). The mobile phase consisted of Water (A) and acetonitrile (B) with 0.1% formic acid and flow rate of 400 μL/min. The elution gradient was adjusted as follows: 5% B (0 min), 5% B (1 min), 70% B (20 min), 100% B (24 min), and 100% B (27 min). One μL of samples were injected and analysis was made in positive ion mode. The conditions of the ESI source were similar to a previous study [29].

### In vivo experiments

#### Acute oral toxicity in rats

Acute oral toxicity of C2RLP was conducted in six 6-week old female Sprague-Dawley rats (average weight of 150 g) purchased from Orient Co. Seoul, South Korea (Charles River Technology). The experiment was carried out according to OECD guidelines - 425. C2RLP was administered at a single oral dose of 2000 mg/kg to three rats, while the remaining three served as untreated controls. The rats were monitored for changes in body weight, water, and food intake. Rats were also carefully inspected for abnormal signs and symptoms such as changes in the color of the skin and eyes, convulsions, diarrhea, lethargy, and coma for a total of 14 days. Finally, rats were euthanized by carbon dioxide inhalation (flow rate adjusted to 15–30% per minute) and pathological examination was carried out [30].

#### Induction of atopy and treatment of mice

**Fig. 1:**
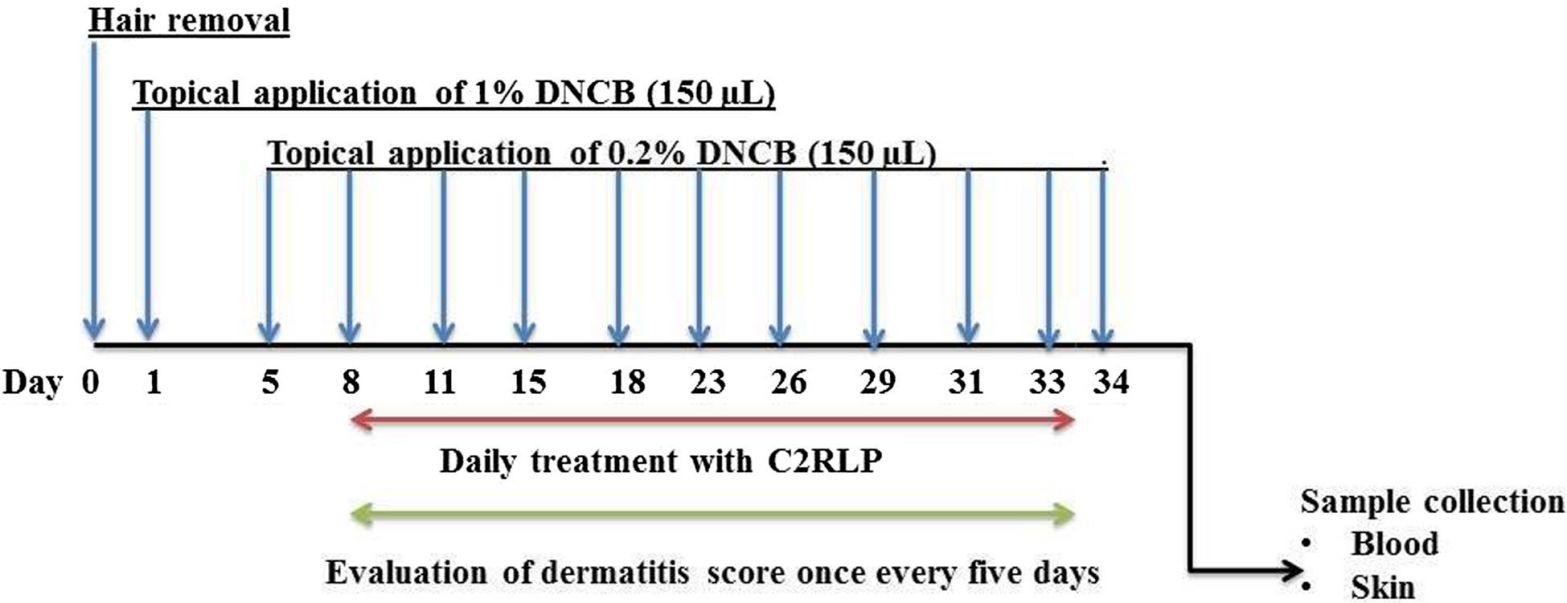
Experimental schedule for induction of atopic dermatitis and treatment with C2RLP in BALB/C mice

Mice were randomly allocated into five groups (*N* = 8) as follows: Group I: treated with petrolatum (Negative control group); Group II: treated with betamethasone (Positive control group); Group III and IV: treated with 2.5 and 5% (*w*/w) C2RLP ointment, respectively and Group V: normal control group. The atopic lesion was induced by using 1-Chloro-2,4-dinitrobenzene (DNCB, Sigma-Aldrich) with slight modifications of previously described methods (Fig. 1) [31, 32]. Immediately on the next day after shaving (day 1), mice were treated with 150 μL of 1% DNCB dissolved in an acetone: olive oil mixture (3:1 vol/vol). Five days after hair removal (day 5), 150 μL of 0.2% DNCB was applied to the shaved area three times a week for almost 4 weeks (until day 33). On the 8th day after hair removal (day 8), mice were treated with C2RLP ointment daily, for 25 days. At the end of the experiment (day 34), mice were euthanized by carbon dioxide inhalation and samples (blood and skin) were collected for chemokine analysis and histopathological examination. Blood was collected using a vacutainer tube and allowed to clot by leaving it undisturbed at room temperature for about 30 min. The clots were removed by centrifuging at 2000x g for 10 min in a refrigerated centrifuge. Serum was stored at -20 °C until analysis. Whereas, skin specimens were fixed in 10% neutral buffered formalin.

#### Evaluation of skin lesion

Skin lesions were recorded for each animal and dermatitis were scored once every five days, as follows [33]: (1) Erythema, (2) skin dryness, (3) Edema, and (4) erosion. Scores of 0 (none), 1 (mild), 2 (moderate) and 3 (severe) were given for each lesion and the individual scores were added to determine the extent of overall dermatitis. A researcher who was unaware of the treatment and control groups conducted the evaluation of dermatitis score.

#### Histopathological investigation

Paraffin-embedded specimens were sectioned to 5 μm thicknesses and subjected to the automated tissue processor. Hematoxylin-Eosin (HE) and Toluidine blue (TB) stained skins sections were examined to determine the degree of epidermal hyperplasia, inflammatory and mast cell infiltration. A pathologist who was unaware of the treatment and control groups performed a microscopic evaluation of skin lesions.

#### Th2 and Th1 cytokines

Serum samples were harvested from blood samples collected at the end of the experiment and the levels of IL-2, IL-4, IL-5, IL-6 IL-13, and IFN-ϒ were analyzed using ELISA kits following the manufacturers’ instructions.

### Statistical analysis

Data are presented as means ± standard deviation (SD). One-way analysis of variance (ANOVA) was conducted by using Prism (GraphPad Software Inc., USA) followed by Tukey’s honestly significant difference (HSD) test. Differences with *P* < 0.05 were considered statistically significant.

## Results

### Effects of C2RLP on nitrite and PGE2 production in RAW 264.7 cells

**Fig. 2:**
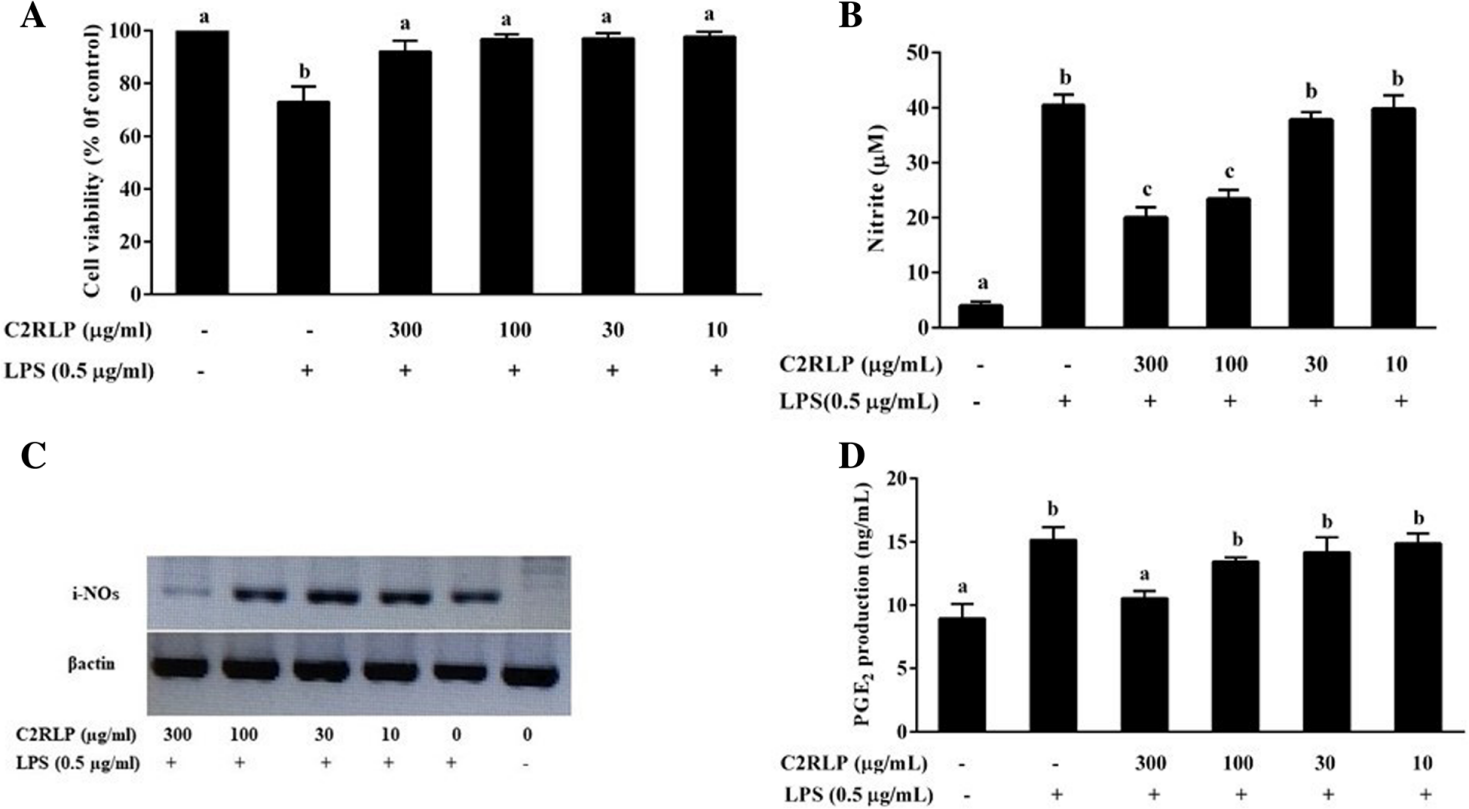
Effects of C2RLP on viability (**a**), NO production (**b**), iNOS-mRNA expression (**c**), and PGE2 production (**d**) of LPS stimulated RAW 264.7 cells. The data shown represent the means of three independent experiments and bars with different letters indicate significant difference (*P* < 0.05)

MTT assay was used to evaluate the effect of C2RLP on the viability of RAW 264.7 cells with an initial concentration of 1 mg/ml. The result demonstrated that > 85% of the cells were alive at the tested concentrations (Fig. 2a). Cells were stimulated with LPS (0.5 μg/ml) prior to treatment with various concentrations of C2RLP, and the levels of NO and PGE2 production were measured after 24 h. LPS stimulated cells produced a higher amount of nitrite (40.5 ± 0.7 μM) (*P* < 0.05) with respect to the non-stimulated cells (Fig. 2b**)**. However, treatment with 100 and 300 μg/ml of C2RLP significantly reduced (*P* < 0.05) the LPS-induced nitrite production. In accordance with the findings in the NO assay, a significant suppression of iNOS gene expression was observed following treatment with C2RLP (Fig. 2c). On the other hand, the level of PGE2 in the cell culture supernatant of LPS-stimulated cells (15.1 ± 0.4 ng/ml) reduced significantly (*P* < 0.05) only in 300 μg/ml C2RLP treated cells with a mean concentration of 10.5 ± 0.2 ng/ml (Fig. 2d**)**.

### C2RLP treatment reduced TARC production in HaCa-T cells

**Fig. 3:**
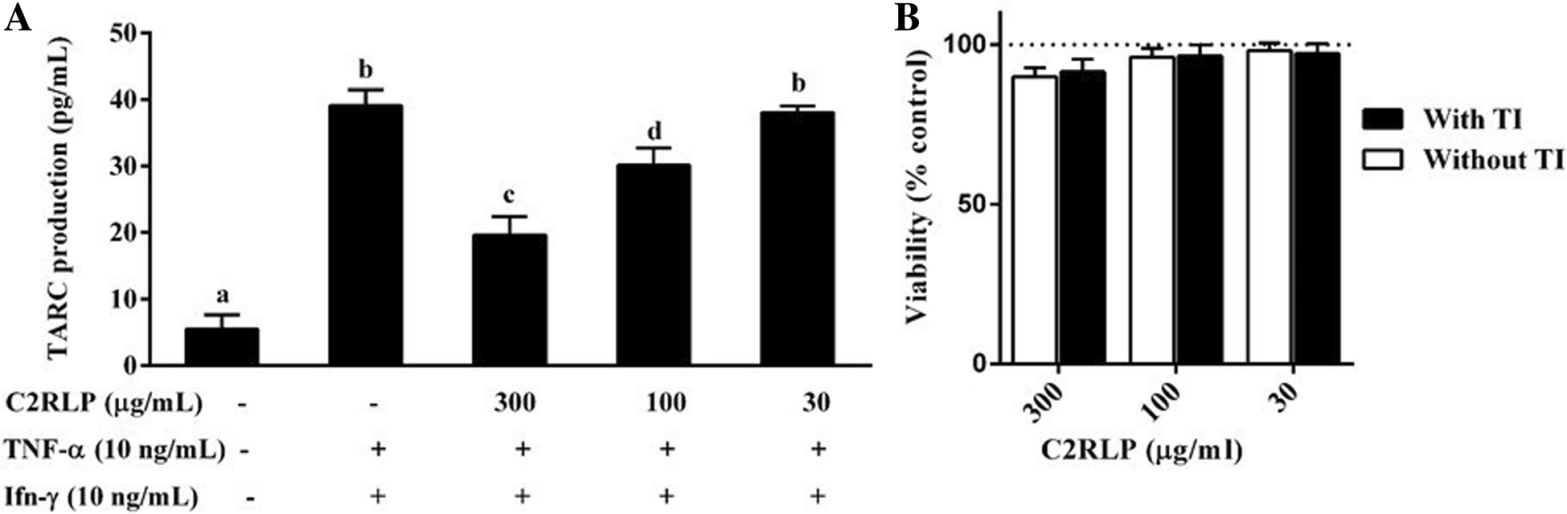
Effects of C2RLP on TARC production (**a**) and viability (**b**) in HaCa-T cells. The data shown represent the means of three independent experiments and bars with different letters indicate significant difference (*P* < 0.05)

HaCa-T cells were stimulated with TI and treated with various concentrations of C2RLP for 24 h. The amount of TARC production in cells stimulated with TI elevated almost 7-fold with respect to the non-stimulated cells (Fig. 3a). The TI induced TARC production was reduced by 49.78 and 22.92% (*P* < 0.05) following treatment with 300 and 100 μg/ml of C2RLP, respectively. In addition, the cytotoxic effect of the test concentrations of C2RLP in HaCa-T cells was assessed prior to determining its effect on TARC production. The results have demonstrated that the test concentrations did not interfere with the viability of HaCa-T cells, both in the presence and absence of TI (Fig. 3b).

### Effect of C2RLP on β-hexosaminidase release in RBL-2H3 cells

**Fig. 4:**
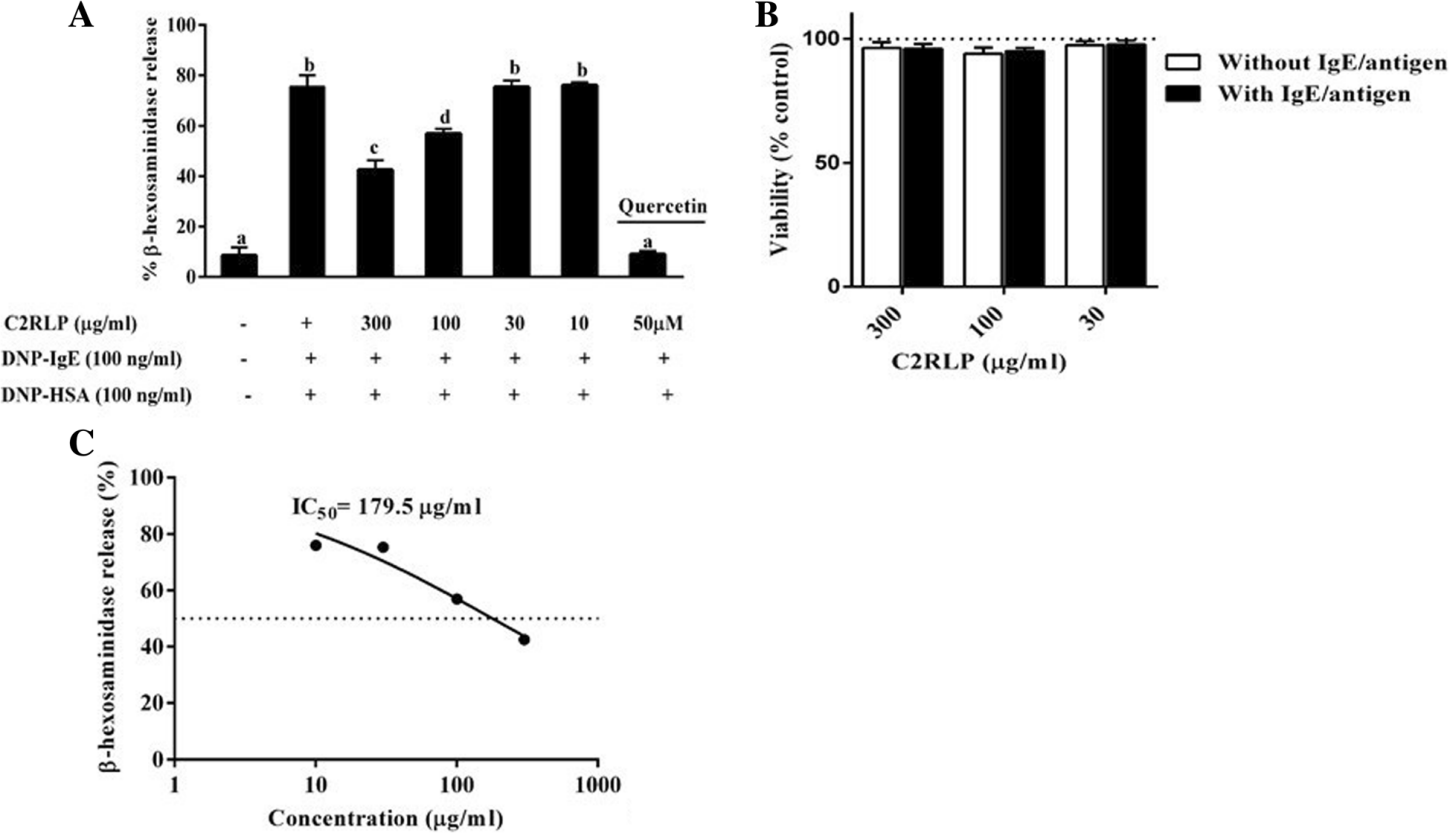
The effects of C2RLP on β-hexosaminidase release (**a**) and viability (**b**) in IgE-DNP antibody sensitized RBL-2H3 cells. The β-hexosaminidase inhibitory activity (IC_50_ value) of C2RLP was also determined (**c**). The data shown represent the means of three independent experiments and bars with different letters indicate significant difference (*P* < 0.05)

The release of β-hexosaminidase from IgE-DNP antibody sensitized RBL-2H3 cells was evaluated to determine the effect of C2RLP on mast cell degranulation. C2RLP significantly reduced (*P* < 0.05) β-hexosaminidase release at concentrations of 100 and 300 μg/ml (IC_50_ = 179.5 μg/ml) with a percentage inhibition of 43.1 ± 1.9 and 57.5 ± 3.8, respectively. In addition, MTT assay demonstrated that C2RLP was non-toxic in both IgE-DNP stimulated and non-stimulated RBL-2H3 cells (Fig. 4 a,b and c).

### Antioxidant activity of C2RLPC

**Fig. 5:**
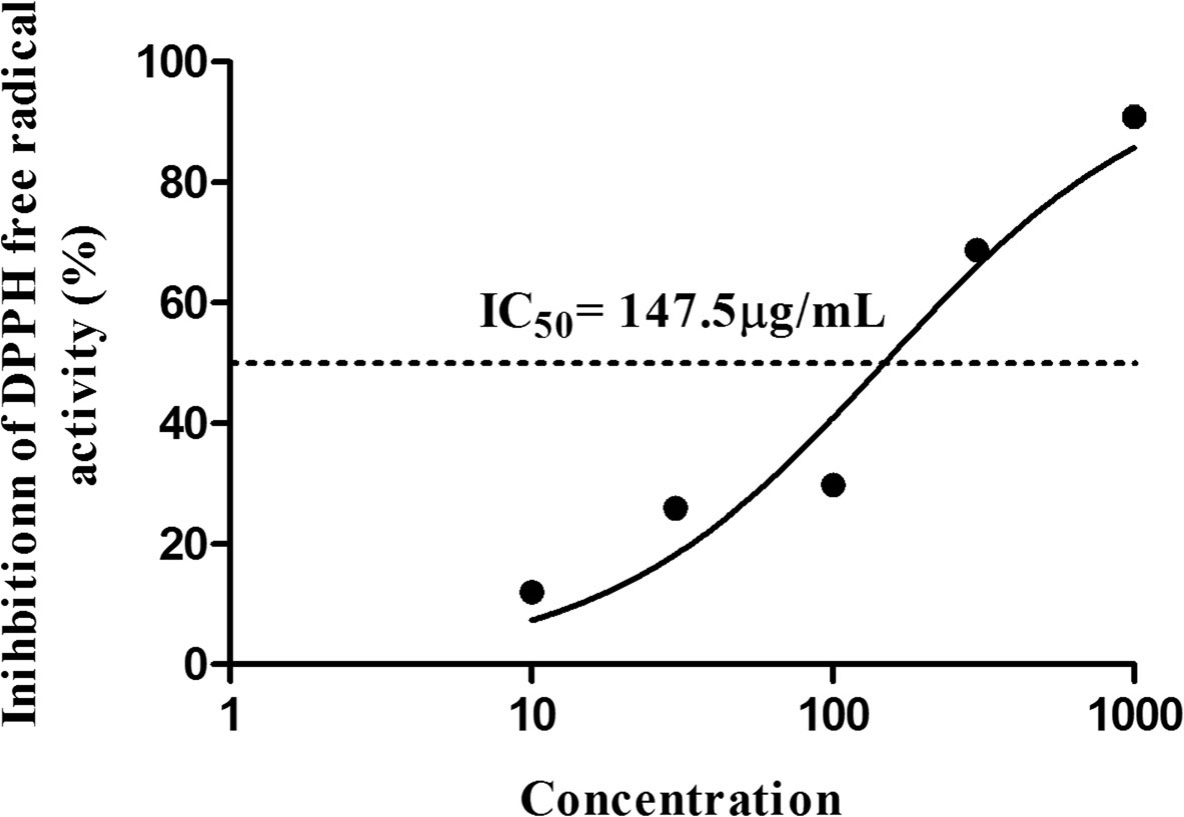
DPPH free radical scavenging activity of C2RLP (IC_50_) following incubation of the C2RLP and DPPH solution at 37 °C for 30 min

The DPPH free radical scavenging assay was conducted to evaluate the scavenging capacity of C2RLP at various concentrations. The result demonstrated a concentration-dependent inhibitory activity of free radicals with an IC_50_ value of 147.5 μg/ml (Fig. 5).

### LC-MS analysis of C2RLP

The main constituents of C2RLP identified by LC-MS are summarized in Table 1. C2RLP possess various compounds that belong mainly to the flavonoids and phenols. Loganin, Ellagic acid, and Kaempferol 3-glucoside were the main compounds identified in C2RLP with retention times of 5.7, 6.4, and 7.5 min, respectively.

**Table 1:** LC-MS analysis of compounds of C2RLP

RT (min)	m/z	△ppm	MS/MS	Formula	Name of compound
3.4	347.1 ([M + H]^+^)	−0.98	183	C14H19O10	Glucopyranosyl methyl gallate
5.7	391.2 ([M + H]^+^)	−0.52	246	C17H27O10	Loganin
6.3	617.1 ([M + H]^+^)	−0.94	303, 315, 599	C28H25O16	Quercetin 3-b-galactoside-2’-O-gallate
6.4	303 ([M + H]^+^)	0.65		C14H7O8	Ellagic acid
6.7	479.1 ([M + H]^+^)	−1.51	303	C21H19O13	Quercetin-3-Glucoronide
7.5	449.1 ([M + H]^+^)	−0.93		C21H21O11	Kaempferol 3-glucoside
8.1	543.2 ([M + H]^+^)	0.18	381	C24H31O14	Cornuside
8.2	433 ([M-H]^−^)		271	C21H21O10	Naringenin7-O-β-D-glucoside
9.5	303.1 ([M + H]^+^)	−0.65	151, 179, 273	C15H11O7	Quercetin

RT, retention time; ([M + H]^+^)/ ([M-H]^−^) base or molecular ions at the positive and negative mode, respectively

### Acute oral toxicity

Oral administration of C2RLP extract was safe up to 2000 mg/kg body weight. None of the rats showed any signs of toxicity such as weight loss, restlessness, ruffled hair, lacrimation, diarrhea, and convulsion.

### Effect of C2RLP ointment on AD-like skin lesions in mice

**Fig. 6:**
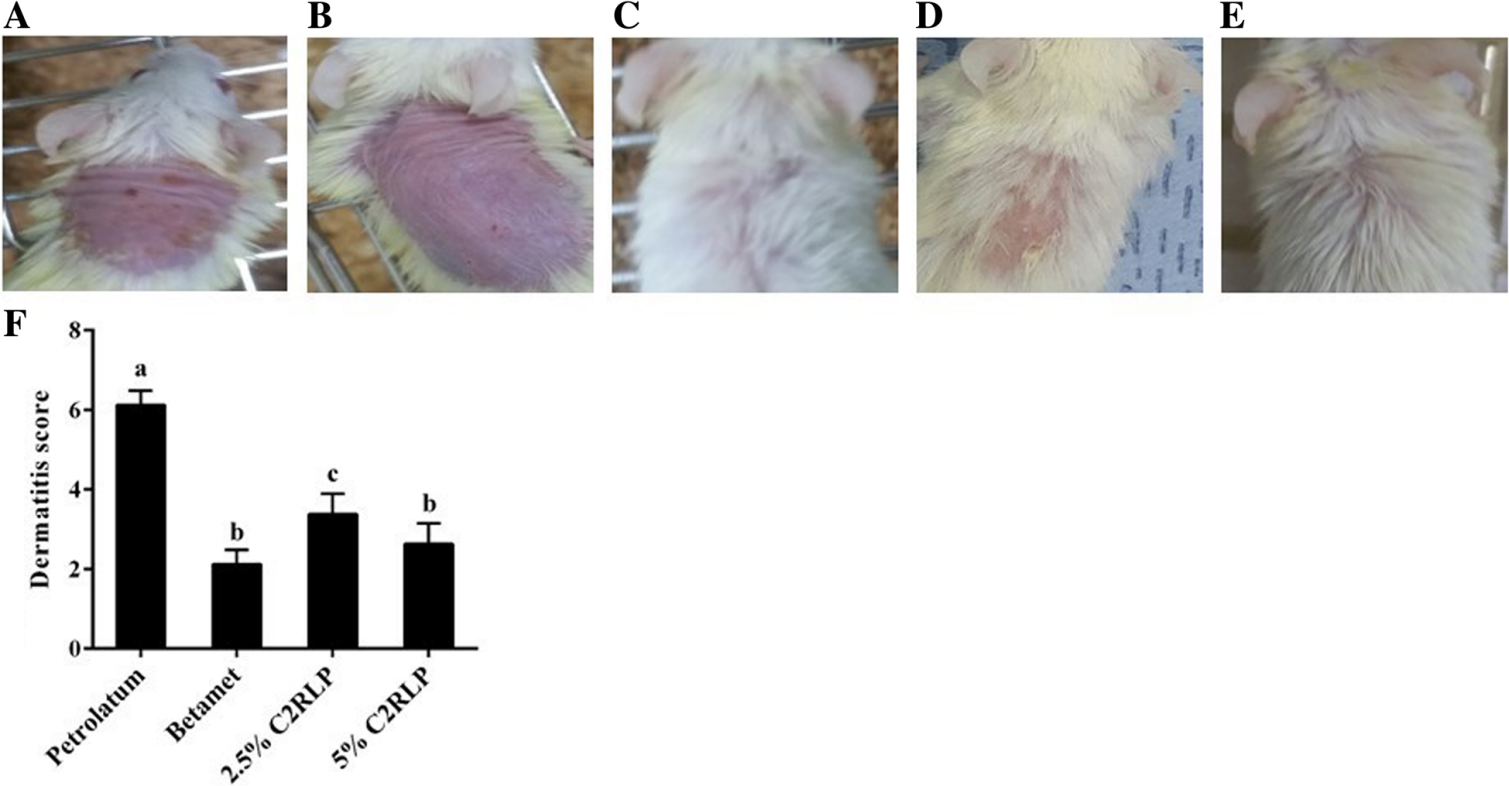
Representative photographs of mouse dorsal skin showing various degrees of alopecia, skin dryness, and erythematous lesions after 2 days of treatment indicated no difference between treatment and control groups (A). Photographs of mice treated with petrolatum (**a**), 2.5% C2RLP (**b**), 5% C2RLP (**c**), and Betamethasone (**d**) indicated treatment with the test substance enhanced the recovery of mice from AD-like lesions compared with petrolatum. Dermatitis score at the end of treatment (day 33) showing a significant difference with respect to DNCB treated control mice (**e**). The data shown in Fig. E represent mean + SD (*n* = 8) and bars with different letters indicate significant difference (*P* < 0.05)

Cutaneous findings related to atopic dermatitis such as mild to moderate erythema, skin dryness, edema, and erosion were evident following application of DNCB for almost 2 weeks (with an average dermatitis score of 6.8) in the petrolatum treated control mice. Topical application of C2RLP and betamethasone significantly suppressed (*P* < 0.05) the cutaneous symptoms as of the 5th and 10th day of treatment, respectively. However, a slight degree of erythema and alopecia were evident in the 2.5% C2RLP treated group. At the end of treatment, the average dermatitis score of the 2.5, 5% C2RLP and betamethasone treated mice were 3.4 ± 0.5, 2.6 ± 0.5 and 2.1 ± 0.4, respectively (Fig. 6 a, b, c, d, e, f). These findings were 2–3 folds lower than the dermatitis score recorded for the petrolatum treated control mice. Therefore, C2RLP produced a concentration-dependent attenuation of DNCB induced AD-like skin lesions in mice.

### Effect of C2RLP on DNCB induced histopathological changes of mouse skin

**Fig. 7:**
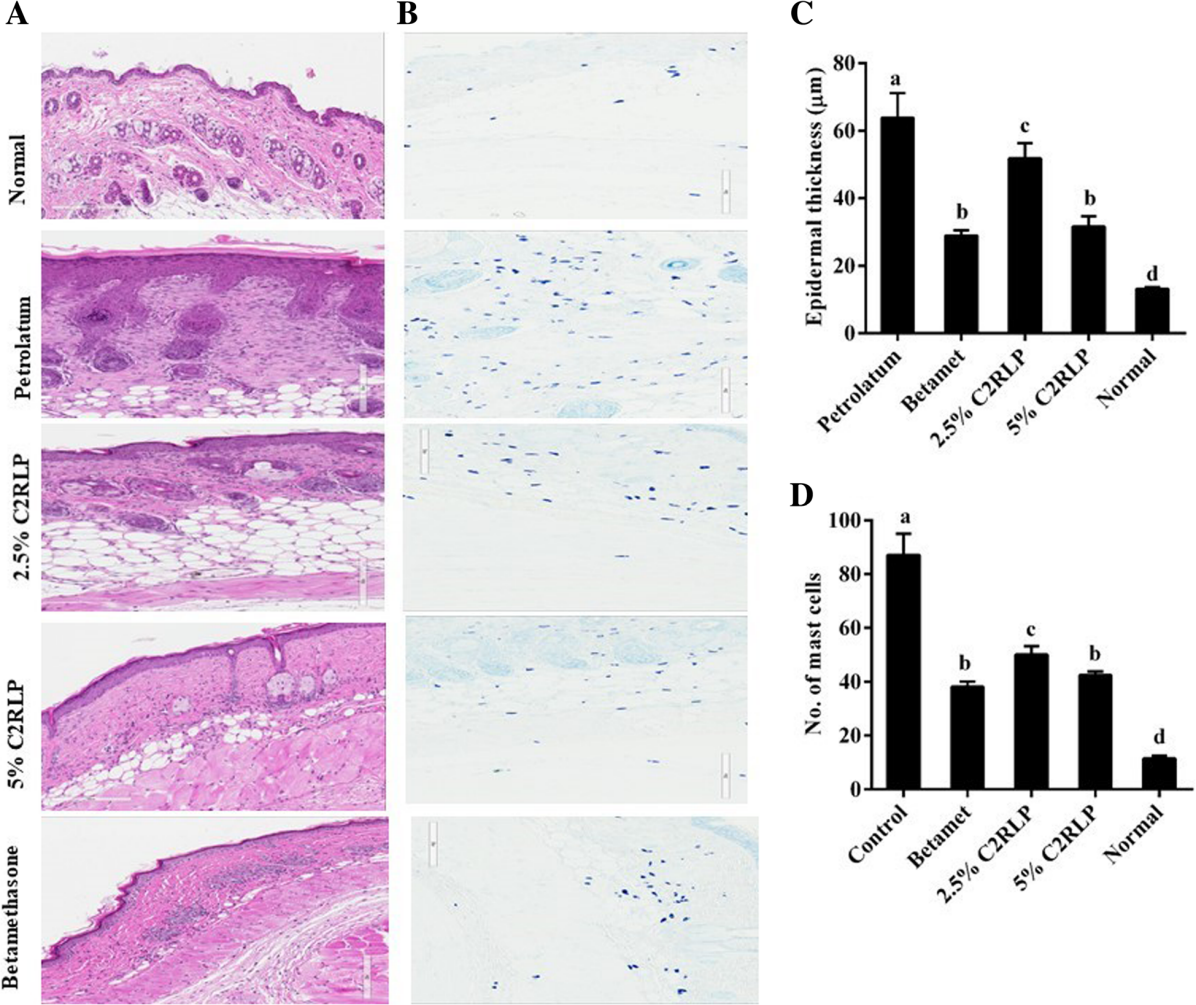
HE (**a**) and TB (**b**) stained skin sections (200x magnification) indicating the effect of C2RLP treatment on the degree of inflammatory cell infiltration and epidermal hyperplasia. Epidermal thickness (**c**) and, the number of mast cells (**d**) was determined in the HE and TB-stained sections, respectively (*n* = 6). The data shown represent the means of three independent experiments and bars with different letters indicate significant difference (*P* < 0.05)

HE-stained skin sections exhibited marked epidermal hyperplasia and inflammatory cell infiltration into the dermal skin layer of the petrolatum treated mice (Fig. 7a**)**. The epidermis of the petrolatum treated control mice were 4.8 fold thicker than the normal control mice. However, treatment with C2RLP and betamethasone exhibited 18.9–54.6% reduction in epidermal hyperplasia and suppressed cellular infiltration compared with the petrolatum treated control. Moreover, the number of mast cells in the dermal layer of the skin was markedly reduced in the C2RLP treated mice with respect to the petrolatum treated mice (Fig. 7 b,c,d).

### Effects of C2RLP on serum levels of cytokines

**Fig. 8:**
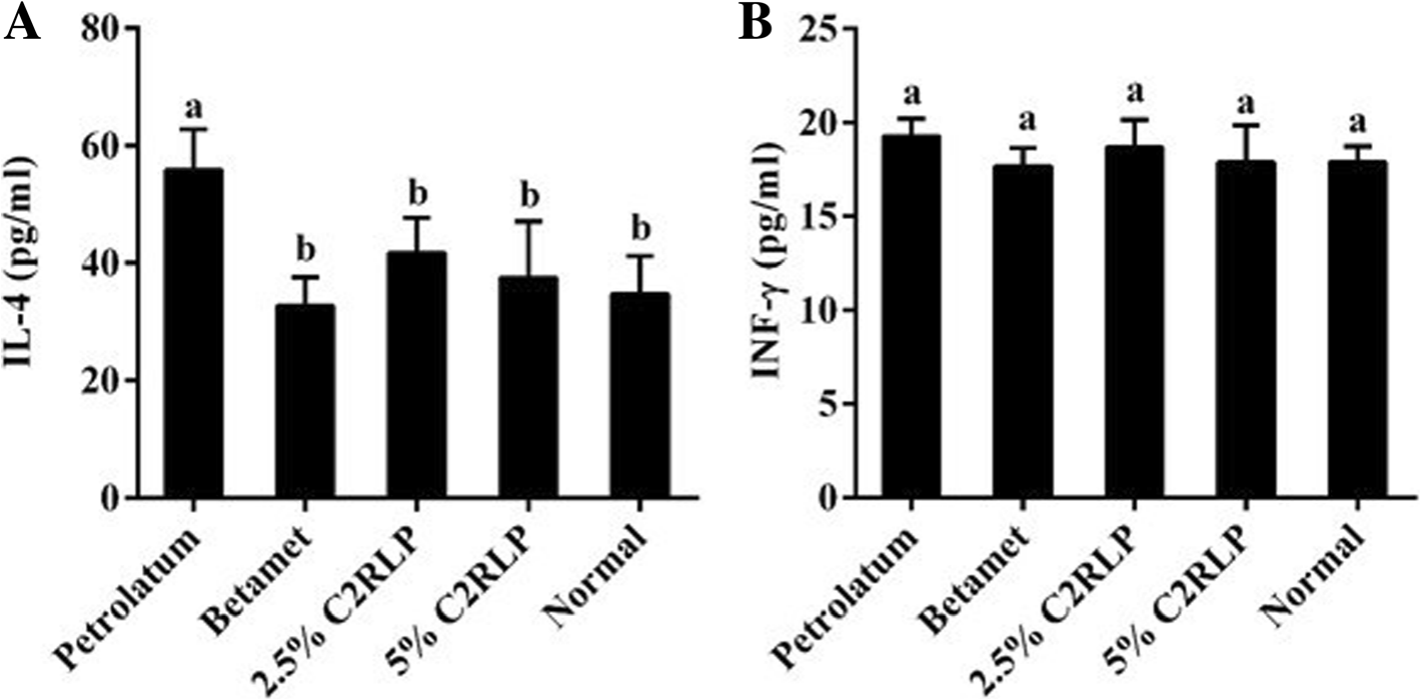
Effects of topical C2RLP treatment on serum levels of IL-4 (**a**) and IFN-γ (**b**) of DNCB treated mice. The data shown represent the mean ± SD (n = 8) and bars with different letters indicate significant difference (*P* < 0.05)

The serum levels of IL-4 in the 2.5 and 5% C2RLP ointment treated groups were 41.7 ± 2.5 and 37.6 ± 3.9 pg/ml, respectively. C2RLP reduced the serum levels of IL-4 in a dose-dependent manner with respect to DNCB treated control group. However, treatment did not produce a significant change (*P* > 0.05) in the level of IFN-ϒ among treatment groups (Fig. 8 a and b). In our study, the levels of IL-2, IL-5, IL-6 IL-10, and IL-13 were not detectable in the serum of Balb/C mice (data not shown).

## Discussion

AD results in various histopathological and pathophysiological changes in mice, which are related to alterations in the levels of proinflammatory cytokines, IgE, and histamine [4, 34]. These changes resulted in epidermal hyperplasia, inflammatory cell infiltration, erythema, alopecia, skin dryness and hyperkeratosis [35] which were clearly observed in petrolatum treated control mice shown in Figs. 6 and 7. The binding of antigen activates infiltrated cells to secrete chemical mediators such as histamine, proteases, cytokines, and chemokines that are essential in the progression of dermatitis [36]. Our study showed that C2RLP treatment suppressed the DNCB induced AD-like lesions and histopathological changes, including epidermal hyperplasia and inflammatory cell infiltration.

The Th1/Th2 cytokine imbalance is vital in the progression of atopic dermatitis, with increased production of IgE and mast cell activation in Th2-dominant AD [37]. Studies have shown that compounds isolated from plants modulate the Th1 and/or Th2 cell response and prevents the development of AD in mice [31, 38]. More precisely, Th2 cytokines such as IL-4, IL-5, IL-13, and IL-4 mediated increment of serum IgE and mast cells were reported in mice with symptoms of AD [2, 39]. In this study, C2RLP significantly reduced the serum levels of IL-4 with respect to petrolatum. IL-4 is known to activate mast cells by inducing isotype switching to IgE synthesis by B cells. The binding of IgE with allergens activates the immune system and induces degranulation [40]. In contrast, C2RLP did not affect the DNCB induced production of IFN-γ which are strong inhibitors of IgE synthesis and Th2 cell proliferation [2]. Therefore, down-regulation of Th2 immunity could be considered as a possible mechanism for the mechanism of C2RLP against AD.

AD is commonly associated with marked infiltration of the skin by mast cells, eosinophils and macrophages [2]. Macrophages are known to release proinflammatory mediators such as NO and PGE_2_, which aggravate the inflammatory responses [41]. Regulation NO production and iNOS expression might be essential because it is known to affect the pathogenesis of several inflammatory diseases, including AD [42]. In the current study, C2RLP inhibited the LPS induced production of NO and PGE_2_ in RAW 264.7 macrophage cells. In addition, C2RLP produced a dose-dependent attenuation of iNOs-mRNA expression.

Chemokines produced by keratinocytes can cause an imbalance in Th1/Th2 cytokines and contributes to the development of atopic lesions [4]. The expression of TARC by keratinocytes in AD patients and in mice with atopic lesions was confirmed in previous studies. TARC is known to attract Th2 cells and aggravates the pathological changes related to AD [41, 43, 44]. In this study, the production of TARC by TI-sensitized HaCa-T cells was reduced following treatment with 300 and 100 μg/ml of C2RLP consolidating the Th2 cell suppressing effects of C2RLP.

Degranulation of activated mast cells and release of mediators are suggested in allergic reactions associated with AD. Mast cell degranulation can be determined by measuring the amount of β-hexosaminidase released from various cell lines including RBL-2H3 [45]. Inflammatory mediators are released from degranulated mast cells following an Fc epsilon RI (FcεRI) receptor activation, which is a high-affinity IgE receptor [46]. In our study, C2RLP exhibited a concentration-dependent inhibition of β-hexosaminidase release from RBL-2H3 cells (IC_50_ = 179.5 μg/ml) with significant inhibitory activity at 300 μg/ ml. Direct inhibition of FcεRI cascade could be one of the mechanisms of the anti-atopic activity of C2RLP.

Oxidative stress in AD is associated with an increase in lipid peroxidation and reduction in the levels of antioxidants. It promotes tissue inflammation through upregulation of genes that code for pro-inflammatory cytokines and subsequent release of free radicals [47, 48]. Oxidative stress can also alter the integrity of epidermal keratinocytes by damaging DNA and cellular enzymes [49]. Previous studies have confirmed higher levels of lipid peroxidation and lower levels of antioxidants in patients with inflammatory skin conditions that resembles AD such as eczema [50] and alopecia areata [51]. Therefore, the antioxidant activity of C2RLP could contribute to the reduction in reactive oxygen species and alleviate the oxidative stress associated with AD.

Chemical compounds are implicated directly or indirectly in the biological effects of most plant extracts. The study revealed the presence of various compounds in C2RLP, mainly Loganin, Ellagic acid, and Kaempferol 3-glucoside (Table 1). Previous studies indicated that suppression of NF-κB and MAP- kinases (mitogen-activated protein kinases) are critical to inhibit the secretion of pro-inflammatory cytokines and reduce the number of mast cells, which are involved in the inflammatory response [52, 53]. Among the major metabolites, loganin is reported to inhibit NF-κB activation and MAP kinase [54, 55]. The polyphenolic compound, ellagic acid, is suggested to have a diverse biological activity, including antibacterial, antioxidant, anti-inflammatory and anti-carcinogenic actions [56]. Most importantly, Ellagic acid has been shown to inhibit activation of MAP kinases [57] and repress NF-κB through down-regulation of the secretion of various inflammatory mediators during AD [58]. Kaempferol-3-O-glucoside and its derivatives were also reported to produce an anti-inflammatory effect through inhibition of the activation of cyclooxygenase (COX-2) and iNOS [59, 60]. In addition to the three major compounds, the antioxidant and anti-inflammatory effects of cornuside [61], naringenin7-O-β-D-glucoside [62], and quercetin [63] could also contribute to the protective effect of C2RLP in the development of AD. Therefore, suppression of AD-like lesions in C2RLP treated mice might be due to the synergistic action of these compounds.

## Conclusions

The study results confirmed the absence of in vitro and in vivo toxic effects of C2RLP. C2RLP attenuates the symptoms of atopic dermatitis in mice by modifying local and systemic inflammation. It produces a marked reduction in dermatitis score and inflammatory cell infiltration; and decreased the production of IL-4, NO, PGE2, and TARC. C2RLP also suppressed β-hexosaminidase release, which is the hallmark of allergic reactions and mast cell degranulation. Therefore, the findings of this study suggest that topical application of C2RLP might be effective in preventing the development of AD. However, detailed studies on the molecular mechanism (s) of C2RLP are needed.

## Abbreviations

AD: Atopic dermatitis
DNCB: 1-Chloro-2, 4-dinitrobenzene
HE: Hematoxylin-Eosin
IFN-γ: Interferon- γ
IgE: Immunoglobulin-E
LC-MS: Liquid chromatography mass-spectrometry
NO: Nitrite
PGE2: Prostaglandin E2
TARC: Thymus and activation-regulated chemokine
TB: Toluidine blue
Th2: T-helper 2
